# On Gutta Percha, or Gum Gettanea, a New Substance Imported from Singapore

**Published:** 1848-09

**Authors:** 


***On Gutta Percha, or Gum Gettanea, a new substance imported from Singapore.*** By Mr. Solly and M. Soubeiran.—A remarkable substance bearing the name of Gutta Percha, and in several respects analogous to caoutchouc, though in others very different to that article, was imported last year from Singapore, and attracted the attention of experimental inquirers. Gutta Percha was first sent in 1843, by Dr. Montgomery, who received from the Society of Arts a gold medal as a reward for the introduction of this substance, which was considered capable of many useful applications in the arts. This gentleman appears not, however to have made known anything of its history, unless that it could be obtained in very great quantities at moderate price, and that it was much used by the natives as a substitute for horn or wood in many instances, particularly in the manufacture of the handles of knives.

These several applications are the result of the property which this substance posesses of being softened by heat and becoming firm and hard on cooling, retaining at the same time the forms in which it has been moulded. In this respect it continues in a state of perfect preservation, and is subject to no rupture on wearing, to such a degree that it is represented to be preferable to buffalo-horn in the uses in which this last substance is employed.

In the Journal of Agriculture of the India Society, Dr. Mouatt states, in his account of the chemical examination of this substance, that he obtained results analogous to those obtained in this country by Mr. Solly. Dr. Montgomery ascertained that Gutta Percha, or Gutta Tuban, according to the Malays, is produced by a large tree which grows at Singapore and the neighbouring districts. According to a note of Mr. White, this tree appears to belong to the natural family of Sapotaceæ or Ebenaceæ. The trunk is straight and tall, about three feet in diameter at the base, with numerous branches. The timber is hard. The leaves are alternate petiolated, oblong, slightly-pointed at the apex, conical at the base, from five to six inches long; the lower surface of brown-red, covered as well as the side and the petiole, with a thick down. The flower axillary, sessile, inserted by fours in a small white calyx, persistent, with six divisions, in double series, the external the larger.

The corrolla monopetalous, six-fid; the lobes one fourth of one inch, and the tubes one-eighth of one inch long; caducous.

Twelve stamens in one single series, equal inserted at the opening of the tube. The filaments equal in length to the lobe of the corolla. The anthers sagittate, fixed by their basis to the filaments. Pollen not abundant. Ovary superior, conical sessile, placed on a disk; six cells, each containing one single *ovulum* suspended by a central axis, filament well-marked.

The mode of preparing Gutta Percha is not known. It appears, nevertheless, that it is procured by felling the tree which produces it. It is probable that the sap is removed in layers in proportion as they dry, and that these layers are then united, so as to form solid masses.

The specimens sent to England are in two forms. One set have the appearance of shreds or cuttings of white leather; others of solid rolls, which being cut across, show the thin layers, the assemblage of which form the entire mass.

These masses are far from being pure. A considerable quantity of wood sawings and other vegetable remains appear in the mass.

Gutta Percha is an opaque white, or dirty white substance, with little or no odour, tasteless, insoluble in water. Its colour depends apparently on the presence of the impurities which it contains; for, when purified by hot water in the manner now to be described, the water acquires a coloured tint, while the substance becomes white or gray. It has a silky fibrous texture, which becomes more distinct when the mass is slightly stretched; it is soft, almost unctuous between the fingers, and at the same time it has great resistance.

At ordinary temperature it is hard, leathery, and slightly flexible, when in thin plates presenting the physical characters of horn. Above 50° it becomes more flexible, slightly elastic, but always retaining its hardness and remarkable resistance. It contracts little when it has been forcibly stretched.

At 65° and onward to 70° this substance becomes soft and very plastic; its tenacity diminishes greatly. In this state several portions may be united with great facility, so as to form one body. It is easily purified by immersing it in hot water, when the impurities are quickly and completely separated, and a solid mass of pure substance is obtained.

When crude Gutta Percha is thus softened in boiling water, or by exposure to heat it may receive all the desired shapes; and by cooling it recovers its original hardness, preserving its figure; consequently it may be employed in forming casts of medals, which have all the perfection of those formed by sulphur, without the fragility of these last.

When the substance is hot it may be easily cut by a knife; but when it becomes hard and cold, it gives great resistance even to the saw, like hard caoutchouc. The division of this substance becomes more easy, however, by using a wet instrument, as is done with caoutchouc.

Gutta Percha is much lighter than water. When it is carefully deprived of the water which it contains, it is siill much lighter than water, but much denser than caoutchouc. Its specific gravity is 0.9791, while that of ordinary caoutchouc is 0.9355. When Gutta Percha is subjected for some time to a temperature of 150°, it disengages a small quantity of water, and loses its white aspect, becoming of a deep-gray colour, and translucent. It very soon recovers its original appearance if kept for a short time in hot water, and even in cold water.

In chemical characters it closely resembles caoutchouc, with which it is identical in composition. From this it differs slightly in the manner in which it is acted on by certain liquids, but it differs chiefly in physical properties. Subjected to distillation by fire, it is converted almost entirely into several volatile or gaseous hydro-carbonated substances, which possess almost the same odour and probably the same composition as the volatile oil of caoutchouc. It leaves a scanty carbonaceous residue.

Warmed in a platinum vessel, it melts, forms a sort of froth, and burns with a brilliant fuliginous flame, diffusing the odour of the oils of its distillation. When a fragment thus half-burnt is extinguished, the residue is altered and changed into a viscid fluid.

The ordinary solvents exercise little or no action on Gutta Percha. Water, alcohol, alkaline solutions, the muriatic and acetic acids, have no effect on this body. Concentrated sulphuric acid gradually chars it. Nitric acid slowly oxydizes it, forming a yellow resinous matter. By ether, the volatile oils, oil of pit-coal, it is slowly softened in the cold, and imperfectly dissolved by the aid of heat. The true solvent appears to be oil of turpentine, which with great facility forms a clear colourless solution, from which the Gutta Percha may be separated by distillation of the solvent. It is also dissolved by chloroform and and by naphtha.

Its physical properties appear to render Gutta Percha a very proper substitute for leather, for it is exempt from the inconveniences which prevent caoutchouc from being employed in this manner.

M. Soubeiran subjected to various experiments specimens of Gutta Percha received from the Minister of Commerce, and the following are the results :—

Gutta Percha imported by the China mission has the shape of a round loaf a little flattened at the base. It looks at first sight as if it were closed in an investment of skin; but attentive examination shows that this exterior covering is only the substance itself in a highly dried condition. On cutting the leaf in two, we see that it is formed by a matter still soft which has been added at several distinct times, and the different portions of which form layers placed over each other. The consistence is tenacious, and in some degree membranous. The odour is that of old cheese, yet some of the odour of leather is also perceptible.

Gutta Percha M. Soubeiran found, like Mr. Solly, to become by heat soft, perfecly plastic, and convertible into all sorts of forms, which are retained with accuracy when the substance becomes cold and firm.

Gutta Percha contains chemically at least five different substances :—

1, Pure Gutta Percha ; 2, a vegetable acid ; 3, caseine ; 4, a resin soluble in ether and an oil of turpentine ; and 5, a resin soluble in alcohol.

The presence of caseine is indicated by the smell of spoiled cheese, which is possessed in a high degree by the article brought from China. This odour was not perceptible in a specimen sent M. Soubeiran from London by the kindness of Mr. Calvert. The vegetable acid is found in the water in which the Gutta Percha has been boiled. Its quantity is very small; it is associated with a little brown extractive matter, which may proceed from the impurities mingled with the vegetable fluid.

Alcohol at 40° removes from Gutta Percha a transparent inodorous resin, a little soft, which is easily dissolved in oil of turpentine and in ether.

When, after several digestions in boiling alcohol, Gutta Percha was subjected to prolonged boiling with sulphuric ether in a proper apparatus, M. Soubeiran obtained from it a small quantity of a white yellowish resin, which is perfectly dissolved in sulphuric ether and oil of turpentine. This resin possesses in a high degree the smell of leather, and to this must be ascribed the leathery smell which is perceived in the raw Gutta Percha of commerce.

Gutta Percha, after these two processes by alcohol and ether, loses only a very minute portion of its weight. The substance then consists of a peculiar matter very analogous to caoutchouc, and which is pure Gutta Percha. To deprive it of the impurities which adhere to it, solution by rectified oil of turpentine answers best. The solution allowed to rest becomes quite limpid; and when decanted and precipitated by alcohol, the Gutta Percha is separated in a soft mass, which preserves all its properties after it has been several times washed in boiling alcohol. In this state it may be dried in a stove; and M. Soubeiran kept it in a stove heated to 100°, where it was softened.

Subjected to definite analysis in a long tube, with a mixture of chlorate of potass and oxide of copper,—after three analytical processes the following results were obtained—

| | |
|---|---|
| Carbon, 83·5 | Water, 11·3 |
| 83·5 | 11·6 |
| 83·4 | 11·5 |

As it was ascertained that the matter left no trace of cinder it follows that it contained a little oxygen. This M. Soubeiran ascribes to the impossibility of separating completely the Gutta Percha from the two resins with which it is associated.

The composition of Gutta Percha, calculated on the results of the foregoing analysis, is—

| | |
|---|---|
| Carbon 12 parts, | 87·8 |
| Hydrogen 20 | 12·2 |

which approaches very closely to the composition of caoutchouc, in which Faraday found

| | |
|---|---|
| Carbon, | 87·2 |
| Hydrogen, | 12·8 |

M. Soubeiran states that he is inclined to believe in the identity of these two substances, though analysis does not pronounce in a positive manner.

Gutta Percha he, nevertheless, admits, is very distinct from caoutchouc as an immediate principle. Its physical properties enable it to perform in industry and the arts, an important part;—the manufacture of whips, impermeable or waterproof soles to boots and shoes, handles of tools and instruments, and a multitude of domestic utensils. It is further a peculiar character of this material, that all articles made from it will be at the same time resisting and in some degree durable; and when they are once unserviceable in their present form, they require merely to be immersed in hot water in order to refit them or to make them serve in a new form.

Hats manufactured of this substance have been recently announced in London. It is well calculated for making bougies, catheters, probangs, and œsophagus and laryngotomy and lithotomy tubes.—*Pharmaceutic Journal*, *et Journal de Pharmacie.*

